# Cathepsin B- and L-like Protease Activities Are Induced During Developmental Barley Leaf Senescence

**DOI:** 10.3390/plants13213009

**Published:** 2024-10-28

**Authors:** Igor A. Schepetkin, Andreas M. Fischer

**Affiliations:** Department of Plant Sciences and Plant Pathology, Montana State University, Bozeman, MT 59717, USA

**Keywords:** aleurain, barley, cysteine protease, *Hordeum vulgare* L., leaf senescence, protease inhibitor, ribulose-1,5-bisphosphate carboxylase/oxygenase (Rubisco), senescence-associated protease SAG12

## Abstract

Leaf senescence is a developmental process allowing nutrient remobilization to sink organs. Previously cysteine proteases have been found to be highly expressed during leaf senescence in different plant species. Using biochemical and immunoblotting approaches, we characterized developmental senescence of barley (*Hordeum vulgare* L. var. ‘GemCraft’) leaves collected from 0 to 6 weeks after the onset of flowering. A decrease in total protein and ribulose-1,5-bisphosphate carboxylase/oxygenase (Rubisco) large subunits occurred in parallel with an increase in proteolytic activity measured using the fluorogenic substrates Z-RR-AMC, Z-FR-AMC, and casein labeled with fluorescein isothiocyanate (casein-FITC). Aminopeptidase activity detected with R-AMC peaked at week 3 and then decreased, reaching a low level by week 6. Maximal proteolytic activity with Z-FR-AMC and Z-RR-AMC was detected from pH 4.0 to pH 5.5 and pH 6.5 to pH 7.4, respectively, while two pH optima (pH 3.6 to pH 4.5 and pH 6.5 to pH 7.4) were found for casein-FITC. Compound E-64, an irreversible cysteine protease inhibitor, and CAA0225, a selective cathepsin L inhibitor, effectively inhibited proteolytic activity with IC_50_ values in the nanomolar range. CA-074, a selective cathepsin B inhibitor, was less potent under the same experimental conditions, with IC_50_ in the micromolar range. Inhibition by leupeptin and phenylmethylsulfonyl fluoride (PMSF) was weak, and pepstatin A, an inhibitor of aspartic acid proteases, had no effect at the concentrations studied (up to 0.2 mM). Maximal proteolytic activity with the aminopeptidase substrate R-AMC was detected from pH 7.0 to pH 8.0. The pH profile of DCG-04 (a biotinylated activity probe derived from E-64) binding corresponded to that found with Z-FR-AMC, suggesting that the major active proteases are related to cathepsins B and L. Moreover, immunoblotting detected increased levels of barley SAG12 orthologs and aleurain, confirming a possible role of these enzymes in senescing leaves.

## 1. Introduction

Leaf senescence is the final stage of leaf development, ending in large-scale programmed cell death [1,2]. It is not only a catabolic process, but also a recycling one in which nutrients are translocated from the senescing tissues to young leaves, developing seeds, or storage tissues [2,3,4]. Senescence and death of plant organs such as leaves or flowers confer a developmental program in the cells that eventually eliminates a non-functional part as it allows the transport of accumulated nutrients to other growing and developing plant parts [2]. During leaf senescence, chloroplast proteins such as ribulose-1,5-bisphosphate carboxylase (Rubisco) are degraded in preparation for nitrogen remobilization to sink organs [5]. The large subunit of Rubisco is an important protein linked to crop productivity [6].

Almost all protease families have been associated with plant senescence [7]. Available data indicate that papain-like cysteine proteases (PLCPs; family C1A in the MEROPS classification system) [8] control bulk degradation of chloroplast proteins in senescing leaves [7,9]. PLCPs are classified as cathepsin L-, B-, H-, and F-like according to their phylogenetic relationships and gene structures [10]. Cathepsin B- and L-like proteases are involved in plant development, germination, senescence, microspore embryogenesis, pathogen defense, and responses to abiotic stress, including programmed cell death [10,11,12]. The genomics and transcriptomics of barley PLCPs (also known as *Hv*Paps) have been previously characterized with upregulation of several *HvPap* genes during leaf senescence [13,14,15,16,17] (Table 1).

However, while several *Hv*Paps including *Hv*Paps-1, -14, and -19 were previously analyzed through molecular and biochemical approaches [18,19,20,21], a comprehensive understanding of *Hv*Pap activities in senescence-associated nitrogen remobilization has not been achieved yet. To advance this line of research, we have analyzed *Hv*Paps using a combination of activity assays, inhibitor studies, activity-based profiling, and immunoblotting. These approaches indicate that cathepsin B- and L-like enzymes are predominantly active in senescing barley leaves.

## 2. Results and Discussion

### 2.1. Total Protein and Rubisco Contents in Senescing Barley Leaves

**Figure 1:**
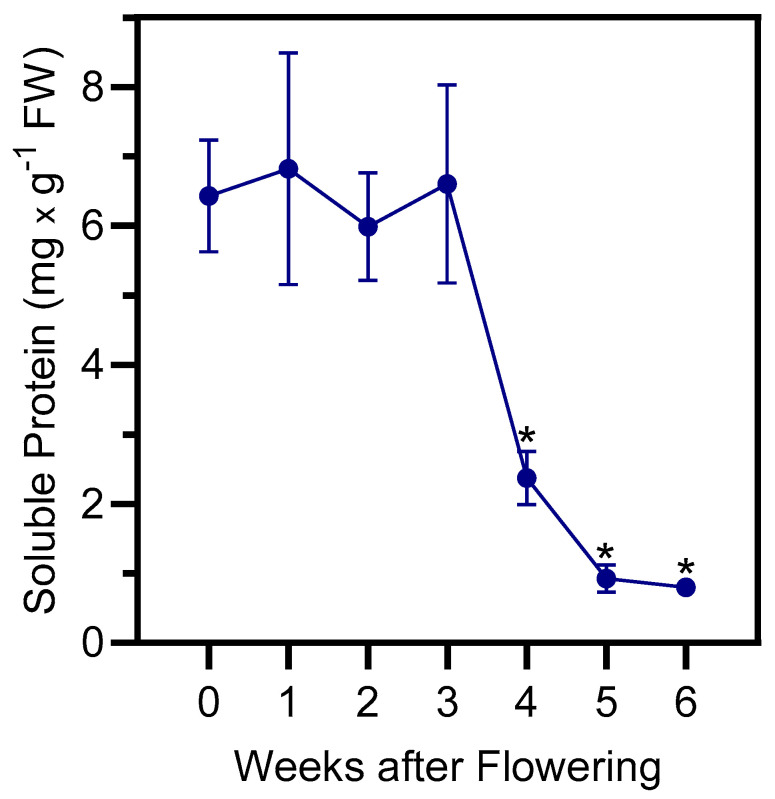
Total soluble protein content was followed over a timespan of 6 weeks, starting with the onset of flowering (0 weeks). Second leaves below the ear were analyzed. The data are presented as the mean values ± S.D. of four biological replicates. Statistically significant differences (* *p* < 0.01) with “week-0” samples are indicated.

Protein degradation, allowing the recycling of nitrogen, is the most important catabolic process that occurs during senescence [7]. Changes in protein levels in barley leaves were examined weekly during a 6-week plant senescence period starting from the onset of flowering (week 0), which occurred 49 days after sowing, and until terminal senescence (week 6). The variety ‘GemCraft’, used in this study [22], has very small flag leaves, prompting us to analyze second leaves below the ear (i.e., leaves directly below flag leaves). In these leaves, protein content significantly (*p* < 0.01) decreased after week 3, reaching very low levels by week 6 (Figure 1).

**Figure 2:**
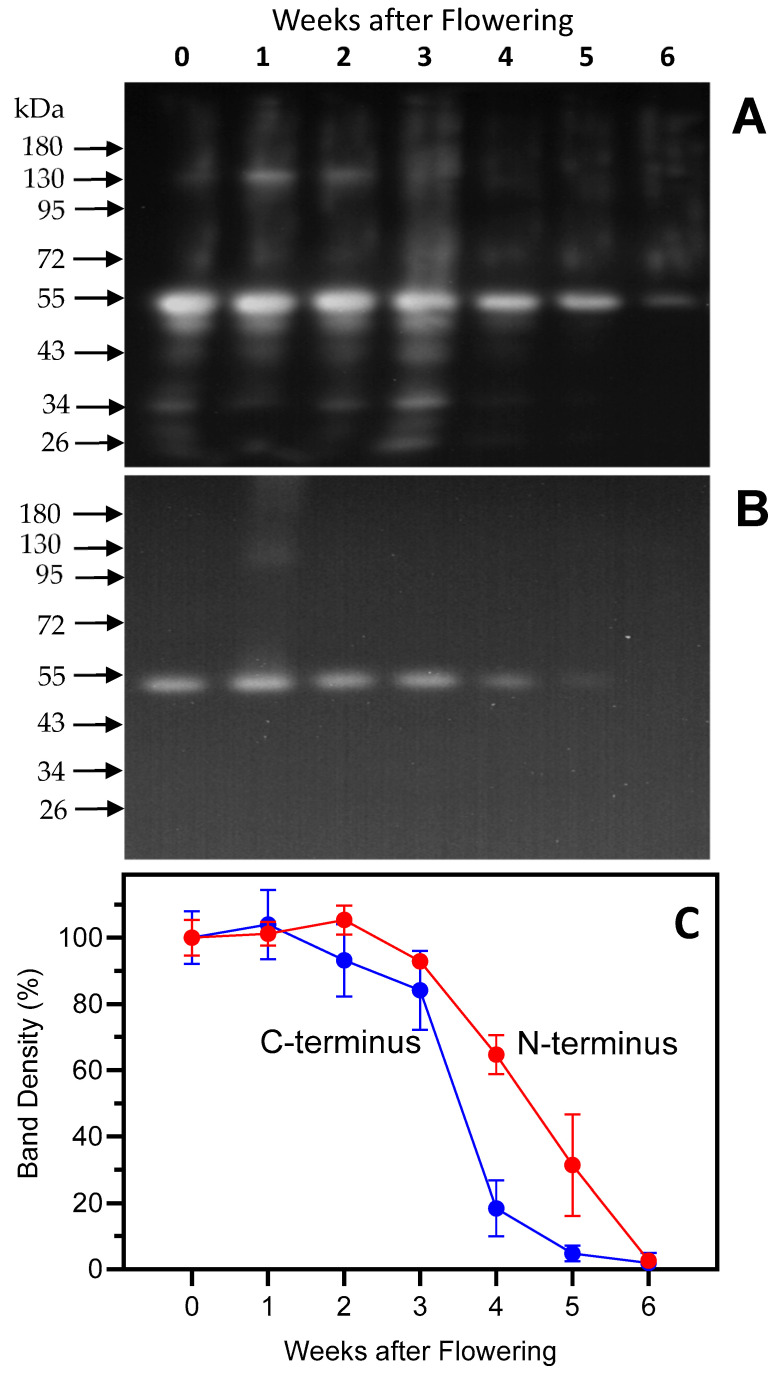
Rubisco LSU contents were followed over a timespan of 6 weeks, starting with the onset of flowering (0 weeks). Second leaves below the ear were analyzed. Rubisco LSU was detected using antibodies raised against the N-terminus (panel (**A**)) or the C-terminus (panel (**B**)). Each lane was loaded with extract obtained from an equal amount of fresh weight (corresponding to 20 μg protein at week 0). Panel (**C**): Densitometry calculations of the 53 kD LSU bands are based on 3 blots for each data point, with bands normalized to week 0 (100%).

Ribulose-1,5-bisphosphate carboxylase/oxygenase (Rubisco) catalyzes the addition of CO_2_ to ribulose-1,5-bisphosphate in the first step of the Calvin cycle. The amount of Rubisco in primary barley leaves has been shown to vary from 15 to 50% of the total soluble protein [23]. A decrease in Rubisco is a good senescence indicator because this protein is the largest source of nitrogen which can be relocalized to the sink organs [24]. A decrease in Rubisco large subunit (LSU) contents was observed in the soluble protein fraction of barley leaves under induced and developmental senescence [13,25]. This effect has also been observed in a variety of other species including potato, wheat, hybrid poplar, and radish [26,27,28,29]. In the present study, to analyze Rubisco degradation in naturally senescing barley leaves, antibodies specific for the N- and C-terminus of the Rubisco LSU were used [13]. After week 3, a decrease in the Rubisco LSU was evident in the protein fraction of the leaves investigated using both antibodies (Figure 2) and correlated with a decrease in total soluble protein (Figure 1).

In addition to strongly visualizing the 53 kDa LSU, the N-terminus peptide antibodies showed several smaller bands with molecular weights of approximately 46.0, 43.0, 35.0, and 26.0 kDa. Visualization of these bands was maximal at 3 weeks, before the onset of rapid protein degradation. Bands with similar molecular weights were previously identified in our lab using the same antibody in senescing barley leaves [13,30].

### 2.2. Total Proteolytic and Cathepsin B/L-like Activities

**Figure 3:**
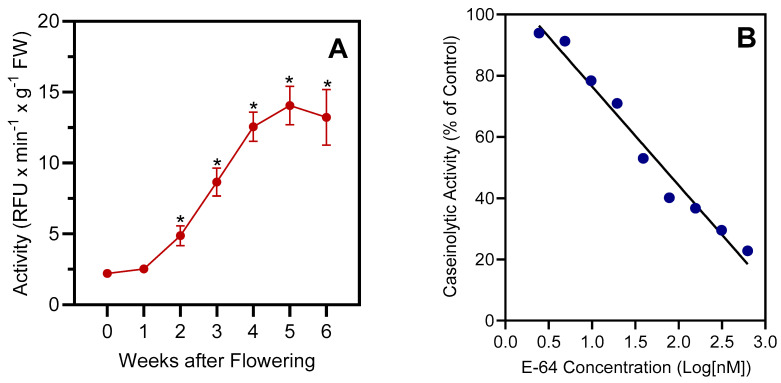
Total proteolytic activity in senescing barley leaves. Panel (**A**): Activity measured with the fluorogenic substrate casein-FITC was followed over a timespan of 6 weeks, starting with the onset of flowering (0 weeks). Second leaves below the ear were analyzed. The data show mean values ± S.D. of four biological replicates. Panel (**B**): Concentration-dependent inhibition of casein-FITC activity by the cysteine protease inhibitor E-64. The experiment was performed three times, with one replicate shown. The activity assays (panels (**A**,**B**)) were performed at pH 7.4. Statistically significant differences (* *p* < 0.01) with “week-0” samples are indicated.

The total protease activity in barley leaves was measured as degradation of casein labeled with fluorescein isothiocyanate (FITC) as the substrate. Casein is degraded by most proteases, including serine, cysteine, and metalloproteases [31]. The increase in fluorescence intensity during degradation of casein-FITC is directly proportional to protease activity and was monitored as a real-time fluorescence signal. Using this substrate, we found that the caseinolytic activity in barley leaf extracts increased strongly from week 0 (onset of flowering) to week 5 (Figure 3A). Compound E-64, an irreversible cysteine protease inhibitor [32], had a potent inhibitory effect on the caseinolytic protease activity of extract obtained from week-4 barley leaves, with IC_50_ = 64.4 ± 3.1 nM (Figure 3B).

**Figure 4:**
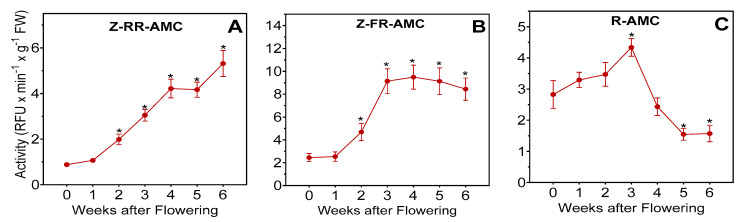
Proteolytic activities in senescing barley leaves. Activities measured with fluorogenic substrates were followed over a timespan of 6 weeks, starting with the onset of flowering (0 weeks). Second leaves below the ear were analyzed. Protease activities were measured with Z-RR-AMC (panel (**A**)), Z-FR-AMC (panel (**B**)), or R-AMC (panel (**C**)). All activity assays were performed at pH 7.4. The data in the panels are presented as mean values ± S.D. of four biological replicates. Statistically significant differences (* *p* < 0.01) with “week-0” samples are indicated.

Because of the high potency of E-64 against caseinolytic activity, two fluorogenic substrates which are specific mainly for cysteine cathepsins were used: Z-FR-AMC to determine cathepsin B- and L-like activity, and Z-RR-AMC to determine cathepsin B-like activity [33,34,35,36]. The Z-RR-AMC cleavage activity increased gradually from non-senescent leaves to week 6 after the onset of flowering (Figure 4A). Proteolytic activity detected by Z-FR-AMC cleavage increased about 3.6-fold between weeks 1 and 3 and then remained constant (Figure 4B). Fluorogenic substrate R-AMC was used to determine aminopeptidase B and cathepsin H-like activity [36]. The R-AMC cleavage activity peaked at week 3 and then decreased, reaching a low level by week 6 (Figure 4C).

**Figure 5:**
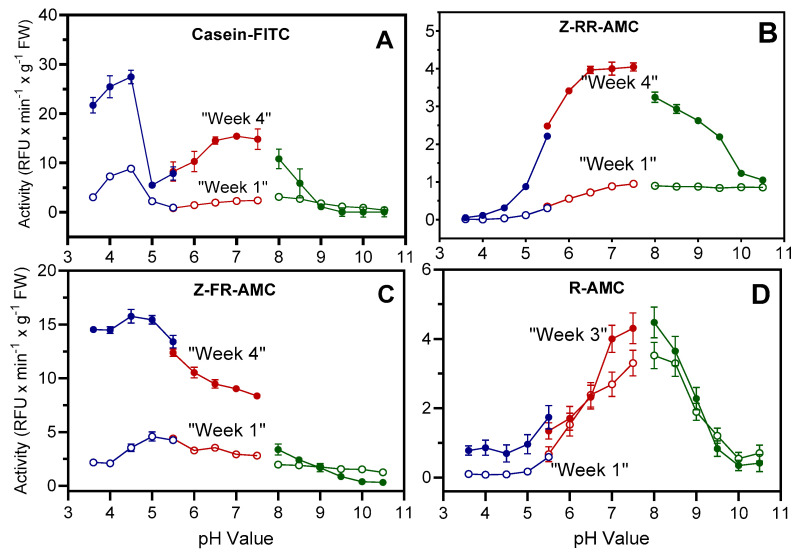
Influence of pH on proteolytic activities in senescing barley leaves. Proteases were extracted from senescing leaves (second leaves below the ear) at 1 week (open circles) and 3 or 4 weeks (closed circles) after the onset of flowering. Activities were measured using the fluorogenic substrates casein-FITC (panel (**A**)), Z-RR-AMC (panel (**B**)), Z-FR-AMC (panel (**C**)), and R-AMC (panel (**D**)). Three different buffers were used with increments of 0.4 or 0.5, including Na-citrate buffer, pH: 3.6–5.5 (blue line); Na-phosphate buffer, pH: 5.5–7.4 (red line); and Tris–HCl buffer, pH: 8–10.5 (green line). The data in the panels are presented as mean values ± S.D. of three technical replicates.

To determine pH optimum curves for enzyme activities hydrolyzing casein-FITC, Z-RR-AMC, Z-FR-AMC, and R-AMC, protein extracts obtained from week-1 and week-4 leaves were used. Two pH optima were found for casein-FITC (3.6–4.5 and 6.5–7.4) (Figure 5A), which may reflect its degradation by a range of proteases. Proteolytic activity peaked between pH 6.5 and 7.4 when using Z-RR-AMC (Figure 5B), while highest activities were found between pH 4.5 and 5.5 for Z-FR-AMC (Figure 5C). The lower pH optimum detected with casein-FITC as compared to Z-FR-AMC may be explained by a change in casein protein structure, resulting in higher availability to proteases at low pH [37,38]. Because highest activities were detected at week 3 with the aminopeptidase substrate R-AMC, extracts from week-3 leaves instead of week-4 leaves were used to determine pH optima. Maximal activities were found between pH 7.0 and 8.0 for both week-1 and week-3 extracts (Figure 5D). Previously, purified cathepsin H-like aminopeptidase (aleurain) from barley leaves showed optimal activities at pH 6.5 to 7.0 [39]. The shift in the optimum pH to 7.0–8.0 observed here may indicate the presence of other aminopeptidases not related to aleurain in the barley leaf extracts.

The effects of protease inhibitors with different specificities on Z-RR-AMC, Z-FR-AMC, and R-AMC cleavage activity in protein extracts of week-4 or week-3 leaf samples were evaluated in detail (Table 2).

Enzyme activities measured at pH 5.5 and 7.4 using the cathepsin B/L substrates Z-RR-AMC and Z-FR-AMC were strongly inhibited by the cysteine protease inhibitor E-64 and by CAA0225, a specific cathepsin L inhibitor [40], with IC_50_ in the nanomolar range. In contrast, the cathepsin B-specific inhibitor CA-074 inhibited proteolytic activity with an IC_50_ in the micromolar range for both substates. Lower sensitivity of a plant cathepsin B-like enzyme to CA-074, when compared with human cathepsin B, has been shown for the enzyme from radish (*Raphanus sativus*) [41]. Recently, Yoon et al. showed that CA-074 binds more favorably to cathepsin B at pH 4.6 than at pH 7.2 [42]. Indeed, the inhibitory activity of CA-074 was higher at low pH for both Z-RR-AMC and Z-FR-AMC substrates. On the other hand, the inhibitory activity of CAA0225 was much higher at pH 7.4. These differences may reflect different pH optima for cathepsin B- and cathepsin L-like activities in the barley extract.

**Figure 6:**
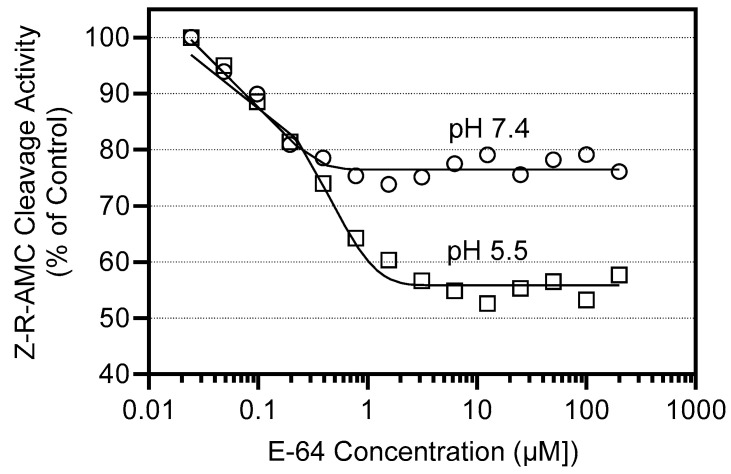
Concentration-dependent inhibition of R-AMC cleavage activity by cysteine protease inhibitor E-64. Proteases were extracted from senescing leaves (second leaves below the ear) at 3 weeks after the onset of flowering. Aminopetidase activity was measured using the fluorogenic substrate R-AMC at pH 5.5 or 7.4. The data show one experiment that is representative of three independent experiments.

Week-3 leaf samples were used to evaluate the effect of protease inhibitors on R-AMC cleavage activity. Inhibitors CAA0225 and CA-074 showed no activity at concentrations up to 0.2 mM with the aminopeptidase substrate, although E-64 inhibited R-AMC degradation by 20–25% at pH 7.4 and by 40–45% at pH 5.5 over a wide (1–200 µM) concentration range (Figure 6). The higher inhibitory efficiency at low pH may reflect the fact that aleurain exhibits optimal activity at pH between 6.5 and 7.0 [39]. Since E-64 is a potent specific inhibitor of cysteine proteases, our data may indicate that cathepsin H-like protease accounts for ~30–40% of the total aminopeptidase activity in barley leaf extract.

Leupeptin, an inhibitor with broad specificity for cysteine, serine, and threonine peptidases [43], showed low efficiency, with IC_50_ in the millimolar range for Z-RR-AMC cleavage activity (Table 2), although this inhibitor was completely inactive with R-AMC. This result is in contrast to the data of Holwerda and Rogers [39] that 10 μM leupeptin inhibited aleurain by ~75%, suggesting again that aleurain contributes only a fraction of total aminopeptidase activity in barley leaf extracts.

Phenylmethylsulfonyl fluoride (PMSF), a serine protease inhibitor, showed low inhibitory activity in Z-RR-AMC cleavage assays, achieving 30% inhibition at a concentration of 2 mM, although it was inactive with Z-FR-AMC and R-AMC.

Bestatin, an inhibitor of leucine aminopeptidase and aminopeptidase B [44], showed inhibitory activity at submillimolar concentrations (IC_50_ = 0.58 mM) with R-AMC substrate. In comparison, for the complete inhibition of aminopeptidase in barley and alfalfa extracts, a bestatin concentration of 0.1 mM was required [45,46]. 1,10-Phenanthroline, a metal chelator, inhibited R-AMC cleavage activity with IC_50_ = 0.56 mM, although this compound was less potent in experiments with Z-RR-AMC and Z-FR-AMC. These data suggest that a substantial fraction of R-AMC cleavage activity in barley leaf extracts is caused by metallo-aminopeptidases.

Finally, pepstatin A, an inhibitor of aspartic proteases, did not show any activity at concentrations up to 0.2 mM with all three substrates.

### 2.3. Activity-Based Identification of Senescence-Associated Cysteine Proteases

To identify cysteine proteases in senescing barley leaves, an activity-based labeling approach [47] was applied. The method is based on the use of DCG-04, a biotinylated derivative of the E-64 inhibitor, which covalently binds to cysteine proteases and can be detected by streptavidin-horseradish peroxidase (streptavidin-HRP) and a chemiluminescent substrate. This approach has previously been used to identify senescence-associated proteases in *Arabidopsis* [48], wheat [47], and barley [21].

**Figure 7:**
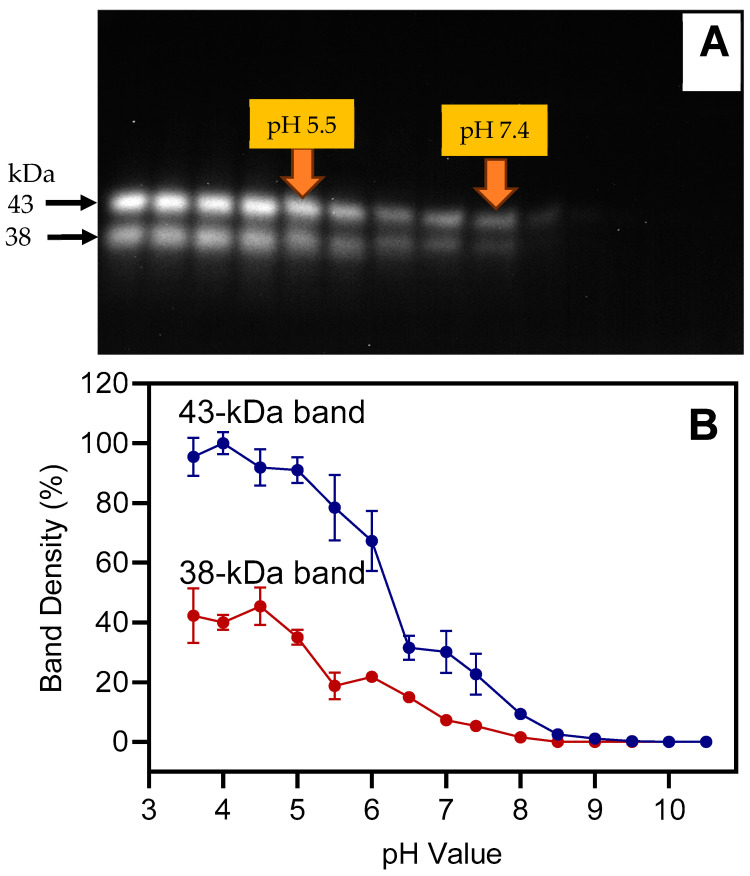
Influence of pH on binding of DCG-04, a cysteine peptidase-specific probe, in barley leaf extracts. Proteins were extracted from second leaves below the ear at 4 weeks after the onset of flowering, with equal amounts of fresh weight loaded in each lane. Proteins were labeled with 2.5 μM DCG-04 for 3 h. Panel (**A**): DCG-04 labeling was detected using streptavidin-HRP and a luminescent substrate. Panel (**B**): Densitometric analysis based on 3 blots (mean values ± S.D.); data were normalized to the 43 kDa band at pH 4.0. Buffers were used with increments of 0.4 or 0.5, including pH: 3.6, 4.0, 4.5, 5.0, 5.5, 6.0, 6.5, 7.0, 7.4, 8.0, 8.5, 9.0, 9.5, 10.0, and 10.5.

Preliminary experiments with Z-RR-AMC and Z-FR-AMC showed that DCG-04 inhibited the proteolytic activity of barley leaf extracts with IC_50_ = 1.8 ± 0.4 μM for both fluorogenic substrates. Based on this value, DCG-04 was used at a concentration of 2.5 μM in the activity-based labeling assays. Because the highest proteolytic activities were found in week-4 barley leaves (Figure 3 and Figure 4), extracts from those leaves were used for subsequent experiments. Extracts were analyzed between pH 3.6 and pH 10.5 with increments of 0.4 or 0.5 pH units. After incubation of the extracts with DCG-04, proteins were separated by SDS-PAGE and the DCG-04-labeled proteases were detected using streptavidin-HRP. Two bands, with molecular weights of ~43 and ~38 kDa, were detected between pH 3.6 and 7.4, with bands barely detectable at more alkaline pH (Figure 7). As expected, the trend seen for these bands corresponds to the pH profile obtained with Z-FR-AMC as a substrate (Figure 5C), indicating that labeling at acidic pH is appropriate.

Previously, five DCG-04-labeled bands, including 48, 44, 35, 32, and 28 kDa, were detected in extracts from senescing barley leaves, with the 32 and 35 kDa bands being most abundant [21]. The observed differences in the patterns of DCG-04-labeled bands could be due to different barley varieties being used (‘Golden Promise’ vs. ‘GemCraft’) and/or to differences in the analyzed leaves (second leaves below the ear, collected after the onset of flowering vs. primary leaves of 21-day-old plants).

**Figure 8:**
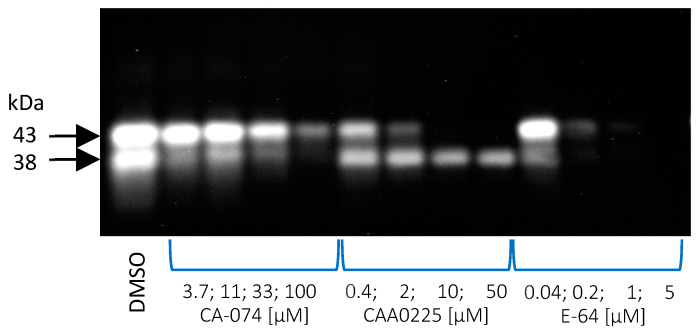
Inhibition of DCG-04 binding by the inhibitors E-64, CAA0225, and CA-074. The protein extracts of barley leaves obtained 4 weeks after flowering were preincubated with different concentrations of the inhibitors and proteins were labeled with 2.5 μM DCG-04 at pH 5.5 for 3 h. DMSO (solvent for inhibitors) was used as a control.

In the next series of experiments, effects of the inhibitors E-64, CAA0225, and CA-074 on DCG-04 binding were evaluated. E-64 inhibited DCG-04 binding to the 38 and 43 kDa bands and completely blocked activity at 1 µM for both bands (Figure 8). CAA0225 totally inhibited binding to the 43 kDa band at a concentration >6 µM but did not block binding of DCG-04 to the 38 kDa band. On the other hand, CA-074 did not totally block the binding to the 43 kDa band, even at a concentration of 100 μM, but was more active toward the 38 kDa band.

**Figure 9:**
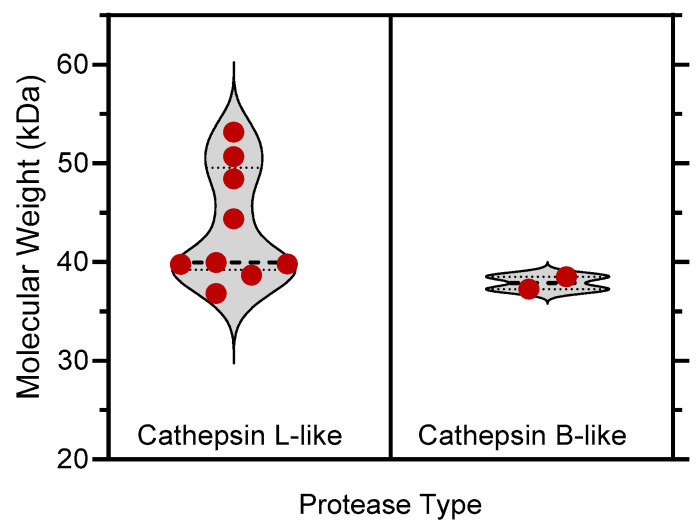
Violin-type plots showing the distribution of molecular weights for full-length cathepsin L- and B-like proteases with reported gene upregulation in transcriptomic analyses of barley leaf senescence [13,14,15,16,17] (see Table 1).

This finding may indicate that the 43 kDa band corresponds to a cathepsin L-like protease or proteases, while the 38 kDa band represents cathepsin B-like protease(s). This assumption also agrees with the molecular weights of full-length cathepsin L- and B-like proteases with reported gene upregulation in the different studies on barley leaf senescence (Figure 9). It should be noted that detailed information on posttranslational modifications (e.g., glycosylation) of these enzymes is lacking; thus, molecular weights of native barley cathepsin B/L-like proteases cannot be predicted based on amino acid sequence only.

**Figure 10:**
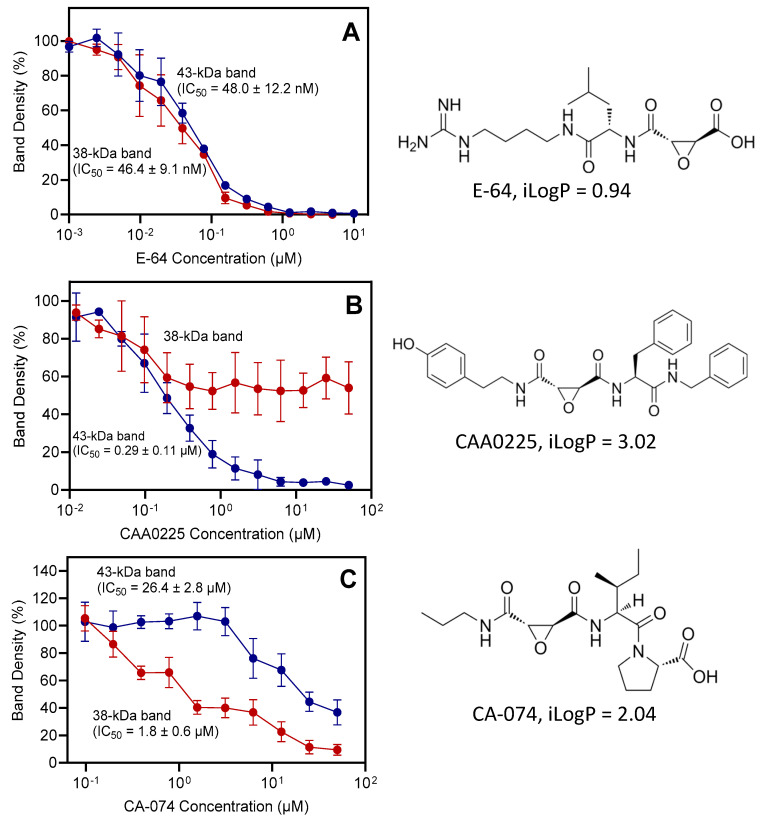
Concentration-dependent effect of the protease inhibitors E-64 (panel (**A**)), CAA0225 (panel (**B**)), and CA-074 (panel (**C**)) on DCG-04 binding to the 38 and 43 kDa bands and IC_50_ values, calculated from the densitometric analysis of blots. Densitometric analysis was based on 2 blots (mean values ± S.D.); data were normalized to the control value (DMSO solvent without inhibitor). The chemical structures of the inhibitors and calculated values of iLogP are shown on the right side of the figure.

An alternative explanation for the opposing effects of the inhibitors CAA0225 and CA-074 on DCG-04 binding to the 38 and 43 kDa bands could be that the 43 kDa band corresponds to the proenzyme form of the labeled protease(s) with high affinity for the relatively hydrophobic inhibitor CAA0225 and low affinity for CA-074 (see calculated values of n-octanol/water partition coefficient iLogP [49] in Figure 10). Indeed, DCG-04 was previously found to bind both mature cathepsin B and its zymogen [50].

IC_50_ values for all three cysteine protease inhibitors on DCG-04 binding to the 38 and 43 kDa bands were calculated from densitometric analysis of blots (blots with the full range of inhibitor concentrations are shown in Supplementary Materials). The found IC_50_ values were in the nanomolar range for E-64 and CAA0225 and in the micromolar range for CA-074 (Figure 10) and were close to the values found in the enzymatic assays (see Table 2).

**Figure 11:**
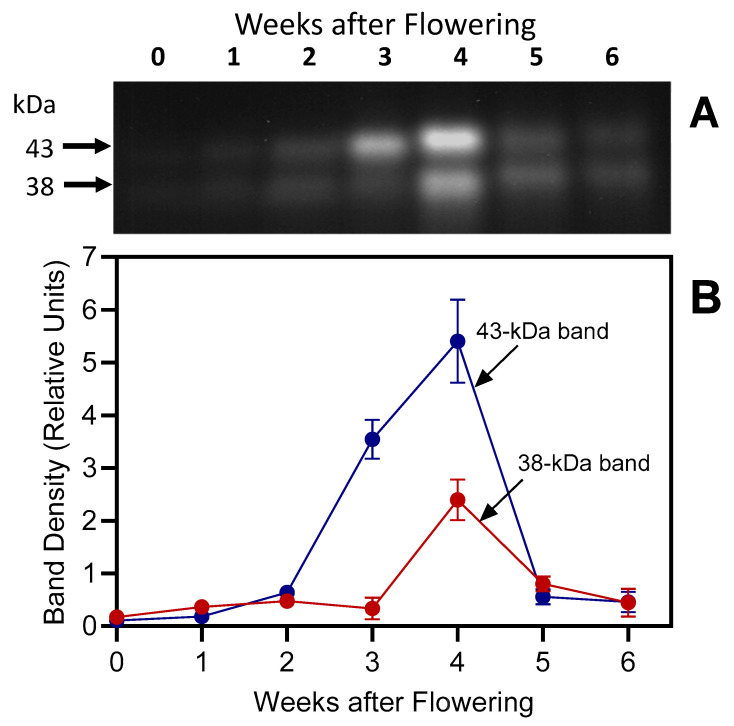
Cysteine peptidase activities in senescing barley leaves. Activities detected by labeling with 2.5 μM DCG-04 (3 h) were followed over a time span of 6 weeks, starting with the onset of flowering (0 weeks). Second leaves below the ear were analyzed, with equal amounts of fresh weight loaded in each lane. Panel (**A**): DCG-04 labeling. Panel (**B**): Densitometric analysis. Calculations were based on 2 blots (mean values ± S.D.).

Because the highest DCG-04 binding was found between pH 3.6 and 5.5 (Figure 7), pH 5.5 was used to monitor cysteine protease activity during natural senescence. The signal increased gradually from week 0 to week 4 and decreased to background level at weeks 5 and 6 after the onset of flowering (Figure 11).

### 2.4. Expression of SAG12 Protein and Aleurain During Senescence

Based on the molecular weights of DCG-04-labeled proteins in barley leaf extracts, several cysteine proteases including cathepsin B-, L-, and H-like proteases may be induced during senescence. In particular, the cysteine protease SAG12 belongs to the cathepsin L-like family, subgroup A and is expressed exclusively during senescence [16,51]. *SAG12* transcript levels have previously been found to increase in barley leaves during induced senescence, and SAG12 is involved in Rubisco degradation [52,53].

**Figure 12:**
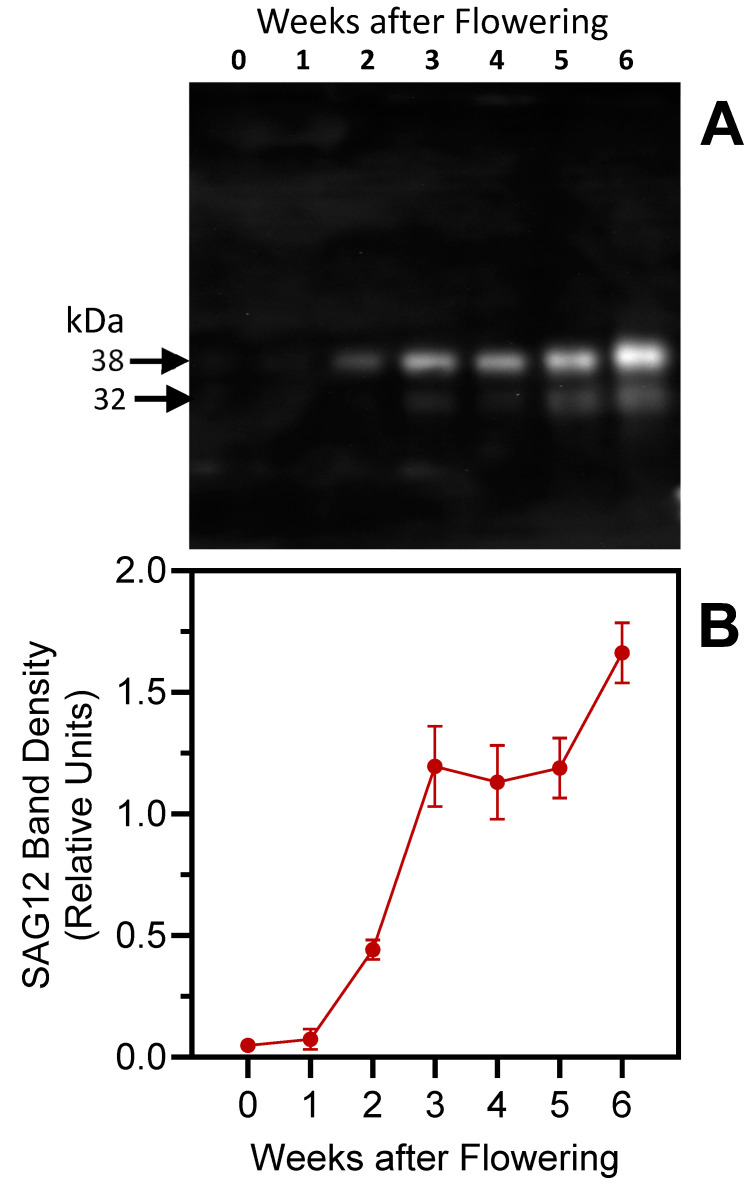
Immunoblot analysis of SAG12 in senescing barley leaves. Panel (**A**): Protein levels were followed over a timespan of 6 weeks, starting with the onset of flowering (0 weeks). Second leaves below the ear were analyzed, with equal amounts of fresh weight loaded in each lane. Panel (**B**): SAG12 densitometric analysis (38 kDa band). Densitometric analyses were performed on 2 blots (mean values ± S.D. are shown).

In our experiments, SAG12-specific antibodies detected a major 38 kDa protein band (Figure 12) in senescing barley leaves (weeks 2–6); the band corresponds to the predicted size of the mature SAG12 protein [54]. The 32 kDa band was not detected in younger leaves and was barely visible in week-5 and week-6 samples. Identical molecular masses (~38 kDa) for the major SAG12 band and the lower of the DCG-04-labeled protein bands (Figure 7, Figure 8 and Figure 11) may indicate that the activity-based approach detects the SAG12 protein. Using the same antibody, Frank et al. [21] have previously identified bands with similar molecular weights in senescing flag leaves of field-grown barley, with increasing levels as senescence progresses.

Among barley PLCPs, *Hv*Pap-17 is most closely related to the *Arabidopsis* SAG12 protease, with ~53% sequence identity. Based on the sequence alignment, the SAG12 antibody may cross-react with *Hv*Pap-17, as well as other barley proteases including *Hv*Pap-9, -10, -11, -14, and -42. Among these, only *Hv*Pap-14 and *Hv*Pap-17 are induced at the transcript level during senescence (see Table 1).

**Figure 13:**
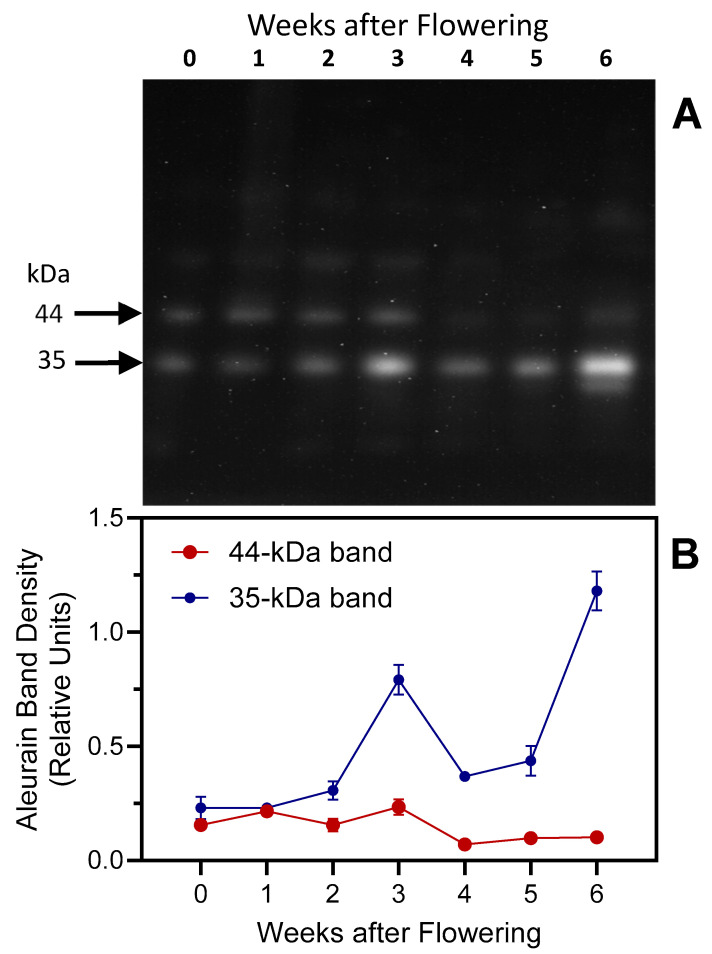
Immunoblot analysis of aleurain in senescing barley leaves. Panel (**A**): Protein levels were followed over a timespan of 6 weeks, starting with the onset of flowering (0 weeks). Second leaves below the ear were analyzed, with equal amounts of fresh weight loaded in each lane. Panel (**B**): Aleurain densitometric analysis. Densitometric analyses were performed on 2 blots (mean values ± S.D.).

Aleurain-like proteins (and their genes) expressed during seed germination [55] and leaf senescence [56,57,58,59], although they may be involved in specific proteolytic events, such as the activation of other enzymes, rather than general protein degradation [39]. Aleurains are orthologous to mammalian cathepsin H proteases [39,60]. The barley aleurain protein is synthesized as a 42 kDa pro-enzyme (pro-aleurain) that is proteolytically processed in a post-Golgi compartment in two steps to form a 32 kDa protein [55]. Using an antibody raised against *Arabidopsis* aleurain, two bands, with molecular weights of ~44 and ~35 kDa, were detected in this study (Figure 13); these molecular weights are identical to those found by Cohen et al. [15] and close to those published by Rogers et al. [55]. Similarly to the aminopeptidase activity assay with the R-AMC substrate (see Figure 4C), the anti-aleurain antibody detected a strong signal in week-3 protein extracts. However, the enzymatic assay likely identified other aminopeptidases besides aleurain, explaining the discrepancy between immunoblotting and activity assays at later dates.

Similarly to results obtained with senescing flag leaves of two other barley genotypes [15], the proenzyme in senescing second leaves of variety ‘GemCraft’ was processed to the mature form between 2 and 3 weeks after the onset of flowering. However, data presented here suggest that the mature form accumulates to higher levels during late senescence stages, with maximal levels detected after six weeks (Figure 13).

## 3. Materials and Methods

### 3.1. Materials and Reagents

E-64 (trans-epoxysuccinyl-L-leucylamido-(4-guanidino) butane), Z-Phe-Arg-7-amino-4-methylcoumarin (Z-FR-AMC), 1,10-phenanthroline, bestatin, protease inhibitor cocktail for plant tissue extracts, leupeptin, dithiothreitol (DTT), and polyvinylpolypyrrolidone (PVPP) were from Millipore-Sigma (St. Louis, MO, USA). CAA0225 ((2*S*,3*S*)-oxirane-2,3-dicarboxylic acid 2-[((*S*)-1-benzylcarbamoyl-2-phenyl-ethyl)-amide] 3-{[2-(4-hydroxy-phenyl)-ethyl]-amide}) was from EMD Millipore Corporation (Burlington, MA, USA). CA-074 [(2*S*)-1-[(2*S*,3*S*)-3-methyl-2-[[(2*S*,3*S*)-3-(propylcarbamoyl)oxirane-2-carbonyl]amino]pentanoyl]pyrrolidine -2-carboxylic acid], an irreversible and specific inhibitor of cathepsin B, was from ApexBio (Boston, MA, USA). Pepstatin A and color-coded prestained protein marker were from Cell Signaling Technologies (Danvers, MA, USA) and PMSF was from Tocris Biosciences (Bristol, U.K.). Streptavidin-HRP conjugate was from GE Healthcare Life Sciences (Northbrook, IL, USA). Casein-FITC was from AAT Bioquest (Pleasanton, CA, USA). Z-Arg-Arg-7-amino-4-methylcoumarin (Z-FR-AMC) was from Echelon Biosciences (Salt Lake City, UT, USA) and Arg-7-amido-4-methylcoumarin (R-AMC) was from AK Scientific (Palo Alto, CA, USA). Dimethyl sulfoxide (DMSO) was from Acros Organics (Fair Lawn, NJ, USA). DCG-04, a biotinylated E-64 derivative [61], was obtained from Psyclo Peptide Inc. (Shanghai, China).

### 3.2. Plant Material and Growth Conditions

Barley plants (*Hordeum vulgare* L. var. ‘GemCraft’) [22] were grown and treated as described previously [14]. Briefly, barley plants (three plants per 4 L pot) were grown in potting soil in a greenhouse bay of the Plant Growth Center at Montana State University (Bozeman, MT, USA) between August and November 2023, with a 22 °C:18 °C day:night temperature cycle. Days were extended to a 16 h photoperiod when required, using Son-Agro 430 W high-pressure sodium lamps (Philips, Somerset, NJ, USA). Plants were fertilized once per week until flowering with Peter’s Professional General-Purpose fertilizer (250 mL per pot; 4 g/L; Scotts-Sierra Horticultural Products Company, Marysville, OH, USA).

The barley variety ‘GemCraft’ has small flag leaves (leaf position directly below the developing ear); second leaves (leaf position directly below the flag leaf) were therefore used in this study. Leaf blades were removed from plants during early afternoon, immediately frozen in liquid nitrogen, and stored at −80 °C until analysis. Each sample consisted of 10 leaf blades, and five samples were collected starting at the onset of flowering, and in weekly intervals, thereafter, using a random sampling approach.

### 3.3. Extraction of Soluble Protein

Frozen leaves were homogenized with a pre-chilled pestle and mortar in cold extraction buffer (0.1 M Tris–HCl, pH 7.4, 2 mM DTT) at a ratio of 167 mg of leaves:1 mL buffer. The homogenate was centrifugated at 20,000× *g* for 30 min at 4 °C. The supernatant, which was kept in an ice bath, was used as a sample for measurements of peptidase activity and protein content.

Protein concentration was determined using Bradford reagent (Thermo Scientific, Rockford, IL, USA) and bovine serum albumin as a standard.

### 3.4. Enzymatic Assays

Fluorogenic substrates Z-FR-AMC to determine cathepsin B- and L-like activity, and Z-RR-AMC to determine cathepsin B-like activity, were used [33,34,35]. These substrates show fluorescence when AMC is released as a consequence of the hydrolysis of the Arg-AMC bond. The emitted fluorescence was detected with a SpectraMax M2 microplate reader (Molecular Devices, San Jose, CA, USA) with λ_ex_ = 360 nm and λ_em_ = 460 nm. Before use, the substrates were dissolved in DMSO at 10 mM and frozen at −20 °C. The measurements were made in 96-well black microplates (PerkinElmer Inc, Waltham, MA, USA), and each well contained a 100 μL final volume of an appropriate 0.1 M buffer with DTT (2 mM), extract (1.6 mg of fresh weight), and substrate (25 μM). The reaction was initiated by the addition of the substrate. The final concentration of DMSO in microplate wells was 1% in all the assays. The assays were conducted at room temperature, and relative fluorescence readings were recorded over a period of 10 min.

Casein-FITC substrate was dissolved in bidistilled water to a concentration of 0.5 mg/mL. The substrate solution (30 µL) and barley leaf extract (70 µL with 3 mg of fresh weight) in an appropriate 0.1 M buffer were mixed in the wells of 96-well black plates and changes in fluorescence (λ_ex_ = 494 nm; λ_em_ = 521 nm) were monitored every 30 s at room temperature for 20 min, again using a SpectraMax M2 microplate plate reader.

For the inhibition assays, an inhibitor (1 µL of stock solution in DMSO) at different concentrations was added to the reaction mixture. For all inhibitors tested, the concentration of inhibitor that caused 50% inhibition of the enzymatic reaction (IC_50_) was calculated by plotting % inhibition against the logarithm of inhibitor concentration (at least six points). The data are presented as the mean values of at least three independent experiments.

### 3.5. Activity-Based Detection of Cysteine Proteases

DCG-04 is a biotinylated derivative of the irreversible inhibitor E-64 which covalently binds to the active site of cysteine peptidases [61]. Protein extracts from barley leaves (67 mg of fresh weight) were incubated with 2.5 μM DCG-04 in 0.1 M citrate buffer, pH 5.5, containing 2 mM DTT. When cysteine protease inhibitors E-64 and CA-074 were used, extracts were preincubated with the inhibitors for 10 min before addition of DCG-04. Labeling was achieved with gentle shaking for 3 h at 37 °C. After the incubation, reactions were stopped and proteins were precipitated with inclusion of 100 mM NaCl, along with 80% acetone for rapid precipitation [62]. The pellets were mixed with Laemmli loading buffer and boiled for 5 min [63]. Proteins were then separated on ready-made ExpressPlus^TM^ 4–12% acrylamide gels (GenScript Inc., Piscataway, NJ, USA) in Tris-MOPS SDS-PAGE Running Buffer (GenScript). After SDS-PAGE, proteins were transblotted onto nitrocellulose membrane (BioTrace™ NT nitrocellulose transfer membrane, Cytiva Life Sciences, Marlborough, MA, USA) and blocked for 1 h in 5% non-fat milk in 20 mM Tris-buffered saline (150 mM NaCl), pH 7.4, containing 0.1% Tween-20 (TBST). The membranes were washed 3 × 10 min in TBST and then incubated with streptavidin-HRP (dilution 1:2000 in 1% non-fat milk in TBST) for 1 h and washed again 3 × 10 min in TBST. The membranes were developed using the SuperSignal™ West Pico PLUS Chemiluminescent Substrate (Thermo Scientific, Rockford, IL, USA) and images were obtained using GeneTools by Syngene on a GeneSys imaging platform (Syngene, Frederick, MD, USA). Luminescent signal intensity was calculated by raw volume using GeneTools analysis Version 4.0 software from Syngene.

To examine the dynamics of DCG-04 binding we performed preliminary time course experiments. Acetone was added after 10 min, 20 min, 40 min, 1 h, 2 h, 3 h, and 4 h of DCG-04 treatment to stop the labeling reaction and precipitate proteins. Maximal levels of biotinylated proteases were found at 3 h.

### 3.6. Rubisco Antibodies

Two antibodies, directed against sequences close to the N- and C-terminus of the wheat (*Triticum aestivum* L.) Rubisco LSU, were used for this study. Complete information on these antibodies and their reactivity toward barley Rubisco LSU is given in reference [13].

### 3.7. Immunoblotting

Proteins were extracted from liquid nitrogen-ground barley leaves, using 25 mM Tris–HCl pH 7.5 containing 1% (*w*/*v*) insoluble PVPP, 2 mM DTT, and protease inhibitor cocktail for plant extracts. The inhibitor cocktail contains 4-(2-aminoethyl)benzenesulfonyl fluoride hydrochloride (AEBSF), 1,10-phenanthroline, pepstatin A, leupeptin, bestatin, and E-64 (Cat # P9599, Sigma-Aldrich, St. Louis, MO, USA) as an extraction buffer. Crude samples were centrifuged (10 min, 20,000× *g*, 4 °C), and supernatants were used for the analysis of soluble proteins. The protein extracts were separated on ExpressPlus 4–12% PAGE Gels (GenScript, Piscataway, NJ, USA) using Tris-MOPS running buffer and transferred to nitrocellulose membranes. The blots were blocked overnight at 4 °C in TBST and 5% non-fat dry milk and probed with antibodies, including a 1:4000 dilution for antibodies against the N- and C-termini of the Rubisco LSU, and 1:2000 dilutions for antibodies against SAG12 and aleurain (both from Agrisera AB, Vännäs, Sweden).

After 1 h incubation with the antibodies, the membranes were washed 3 × 10 min in TBST and then incubated with HRP-conjugated secondary anti-rabbit antibody, diluted 1:50,000 before use (Abcam Inc., Waltham, MA, USA), and washed again 3 × 10 min in TBST. The blots were developed using SuperSignal^TM^ West Pico PLUS chemiluminescent substrate (Thermo Scientific, Rockford, IL, USA). Images were obtained using GeneTools by Syngene on a GeneSys imaging platform (Syngene, Frederick, MD, USA). Luminescent signal intensity was calculated by raw volume using GeneTools analysis software.

### 3.8. Molecular Modeling and Statistical Analysis

The physicochemical properties (iLogP) of selected protease inhibitors were computed using SwissADME (http://www.swissadme.ch, accessed on 22 September 2024) [64]. The results are expressed as means ± standard deviation (S.D). Statistical analyses were performed by the unpaired *t*-test.

## 4. Conclusions

In conclusion, enzymatic assays combined with inhibitor screening, DCG-04 labeling, and immunoblotting suggest that cysteine proteases of the cathepsin B- and L-types substantially contribute to overall proteolytic activity of senescing barley leaves. This interpretation is supported by the fact that the pH profile of DCG-04 binding corresponds to the pH profile found for Z-FR-AMC hydrolysis. Moreover, based on immunoblotting, SAG12 and aleurain (cathepsin H-like proteases) levels increased during leaf senescence. We conclude that the barley ortholog(s) of cathepsin L-like cysteine protease SAG12 also contribute to proteolysis in senescing barley leaves.

## Figures and Tables

**Table 1:** Upregulation of *HvPap* genes during barley leaf senescence.

Gene ^a^	Protease ID by MEROPS ^b^; Common Name/Subfamily	HvPap Designator/ Cathepsin Type ^c^	Gene Upregulation (Fold) ^d^	Mol. Weight, kDa ^e^
5Hr1G082810 5HG0499760	C01.022; glycinain	*Hv*Pap-1/cathepsin F	4.0–5.7	41.02
2Hr1G066680 2HG0159420	C01.022; glycinain C01.A04; At2g21430	*Hv*Pap-2/cathepsin F	2.1–5.2	41.86
5Hr1G045090 5HG0462580	C01.065; XCP1 peptidase	*Hv*Pap-4/cathepsin L	>2	38.66
2Hr1G113460 2HG0204520	C01.064; RD21 peptidase	*Hv*Pap-6/cathepsin L	2.3–4.5	53.15
2Hr1G12144 2HG0212170	C01.A12; At5g43060 C01.064; RD21 peptidase	*Hv*Pap-7/cathepsin L	2.1–2.3	50.68
1Hr1G075530 1HG0076400	C01.A13; CP14 peptidase	*Hv*Pap-8/cathepsin L	2.6–13	48.41
5Hr1G061770 5HG0480100	C01.041; aleurain	*Hv*Pap-12/cathepsin H	>2	39.13
5Hr1G089970 5HG0506230	C01.010; vignain C01.A03; AtCEP1	*Hv*Pap-13/cathepsin L	2.1–7.4	39.94
3Hr1G088270 3HG0304500	C01.A03; AtCEP1 C01.168; endopeptidase-A	*Hv*Pap-14/cathepsin L	4.2–70	39.79
6Hr1G008780 6HG0545500	C01.028; fruit bromelain C01.A03; AtCEP1 peptidase	*Hv*Pap-15/cathepsin L	6.2–8.1	44.38
5Hr1G095580 5HG0511140	C01.104; SPG31-like peptidase	*Hv*Pap-17/cathepsin L	14–17	36.8
4Hr1G010390 4HG0339740	C01.049; cathepsin B, plant	*Hv*Pap-19/cathepsin B	2.2–4.7	37.23
4Hr1G010300 4HG0339730	C01.049; cathepsin B, plant	*Hv*Pap-20/cathepsin B	2.5–19	38.51
1Hr1G004580 1HG0004940	C01.131; WCP2 peptidase	*Hv*Pap-22/cathepsin L	>2	39.75

^a^ Gene designations refer to barley variety ‘Morex’ v1 (2016) and v3 (2021) gene models. V1 gene models (first line for each gene) start with ‘HORVU’ before the first number, and v3 gene models (second line) start with ‘HORVU.MOREX.r3’ before the first number (designating the chromosome in both cases). ^b^ MEROPS peptidase database at https://www.ebi.ac.uk/merops, accessed on 1 September 2024. MEROPS protease names were obtained by BLASTing full-length barley peptidase protein sequences against the MEROPS scan (MPRO) dataset. ^c^ For utilized nomenclature see [16]. ^d^ Significant upregulation (>2-fold) in the different studies [13,14,15,16,17]; ^e^ the molecular weight of the full-length protein was calculated.

**Table 2:** Effect of protease inhibitors on cleavage of Z-RR-AMC, Z-FR-AMC, and R-AMC substrates by crude protein extract from barley leaves.

Inhibitor (pH Value)	Z-RR-AMC	Z-FR-AMC	R-AMC
	IC_50_ ^a^		
E-64 (pH 5.5)	6.2 ± 1.7 nM	12.3 ± 2.8 nM	40–45% inhibition at 1–200 µM
E-64 (pH 7.4)	8.1 ± 1.9 nM	24.5 ± 4.2 nM	20–25% inhibition at 0.2–200 µM
CAA0225 (pH 5.5)	90.6 ± 12.2 nM	120.0 ± 14.3 nM	No inhibition up to 0.2 mM
CAA0225 (pH 7.4)	11.6 ± 3.5 nM	11.7 ± 2.6 nM	No inhibition up to 0.2 mM
CA-074 (pH 5.5)	22.7 ± 5.3 µM	11.3 ± 2.2 µM	No inhibition up to 0.2 mM
CA-074 (pH 7.4)	80.7 ± 12.4 µM	31.0 ± 5.6 µM	No inhibition up to 0.2 mM
Leupeptin (pH 7.4)	0.61 ± 0.03 mM	30% inhibition at 0.4 mM	No inhibition up to 0.4 mM
PMSF (pH 7.4)	30% inhibition at 2 mM	No inhibition up to 2 mM	No inhibition up to 2 mM
Pepstatin A (pH 7.4)	No inhibition up to 0.2 mM	No inhibition up to 0.2 mM	No inhibition up to 0.2 mM
Bestatin (pH 7.4)	No inhibition up to 1 mM	No inhibition up to 1 mM	0.58 ± 0.12 mM
1,10-Phenanthroline	3.1 ± 0.2 mM	8.9 ± 0.3 mM	0.56 ± 0.11 mM

^a^ IC_50_ values are based on three technical replicates. Proteases were extracted from senescing leaves (second leaves below the ear) at 4 weeks (Z-RR-AMC, Z-FR-AMC) or 3 weeks (R-AMC) after the onset of flowering.

## Data Availability

Data are contained within the article and Supplementary Materials.

## Supplementary Materials

The following supporting information can be downloaded at: https://www.mdpi.com/article/10.3390/plants13213009/s1, Figure S1. Concentration-dependent effect of the protease inhibitor E-64 on DCG-04 binding to the 38- and 43-kDa bands. The protein extracts of barley leaves obtained 4 weeks after flowering were preincubated with different concentrations of the inhibitor and proteins were labeled with 2.5 μM DCG-04 at pH 5.5 for 3 h. DMSO (solvent for inhibitors) was used as a control. For the treatment, 2-fold dilutions of E-64 were used (10, 5, 2.5, 1.25, 0.64, 0.32, 0.16, 0.08, 0.04, 0.02, 0.01, 0.005, and 0.0025 µM). Representative blot from three independent experiments. Figure S2. Concentration-dependent effect of the protease inhibitor CA-074 on DCG-04 binding to the 38- and 43-kDa bands. The protein extracts of barley leaves obtained 4 weeks after flowering were preincubated with different concentrations of the inhibitor and proteins were labeled with 2.5 μM DCG-04 at pH 5.5 for 3 h. DMSO (solvent for inhibitors) was used as a control. For the treatment, 2-fold dilutions of CA-074 were used (50, 25, 12.5, 6.4, 3.2, 1.6, 0.8, 0.4, 0.2, and 0.1 µM). Representative blot from three independent experiments. Figure S3. Concentration-dependent effect of the protease inhibitor CA0225 on DCG-04 binding to the 38- and 43-kDa bands. The protein extracts of barley leaves obtained 4 weeks after flowering were preincubated with different concentrations of the inhibitor and proteins were labeled with 2.5 μM DCG-04 at pH 5.5 for 3 h. DMSO (solvent for inhibitors) was used as a control. For the treatment, 2-fold dilutions of CA0225 were used (50, 25, 12.5, 6.4, 3.2, 1.6, 0.8, 0.4, 0.2, and 0.1 µM). Representative blot from two independent experiments.

## References

[1] Lv Z., Zhao W.Q., Kong S.X., Li L., Lin S.Y. (2023). Overview of molecular mechanisms of plant leaf development: A systematic review. Front. Plant Sci..

[2] Lei P., Yu F., Liu X.Y. (2023). Recent advances in cellular degradation and nuclear control of leaf senescence. J. Exp. Bot..

[3] Quirino B.F., Noh Y.S., Himelblau E., Amasino R.M. (2000). Molecular aspects of leaf senescence. Trends Plant Sci..

[4] Lee S., Masclaux-Daubresse C. (2021). Current understanding of leaf senescence in rice. Int. J. Mol. Sci..

[5] Buet A., Costa M.L., Martínez D.E., Guiamet J.J. (2019). Chloroplast protein degradation in senescing leaves: Proteases and lytic compartments. Front. Plant Sci..

[6] Prins A., Orr D.J., Andralojc P.J., Reynolds M.P., Carmo-Silva E., Parry M.A. (2016). Rubisco catalytic properties of wild and domesticated relatives provide scope for improving wheat photosynthesis. J. Exp. Bot..

[7] Roberts I.N., Caputo C., Criado M.V., Funk C. (2012). Senescence-associated proteases in plants. Physiol. Plant..

[8] Rawlings N.D., Barrett A.J., Thomas P.D., Huang X., Bateman A., Finn R.D. (2018). The MEROPS database of proteolytic enzymes, their substrates and inhibitors in 2017 and a comparison with peptidases in the PANTHER database. Nucleic Acids Res..

[9] Roberts I.N., Veliz C.G., Criado M.V., Signorini A., Simonetti E., Caputo C. (2017). Identification and expression analysis of 11 subtilase genes during natural and induced senescence of barley plants. J. Plant Physiol..

[10] Martinez M., Diaz I. (2008). The origin and evolution of plant cystatins and their target cysteine proteinases indicate a complex functional relationship. BMC Evol. Biol..

[11] Coppola M., Mach L., Gallois P. (2024). Plant cathepsin B, a versatile protease. Front. Plant Sci..

[12] Cambra I., Hernández D., Diaz I., Martinez M. (2012). Structural basis for specificity of propeptide-enzyme interaction in barley C1A cysteine peptidases. PLoS ONE.

[13] Parrott D.L., McInnerney K., Feller U., Fischer A.M. (2007). Steam-girdling of barley (*Hordeum vulgare*) leaves leads to carbohydrate accumulation and accelerated leaf senescence, facilitating transcriptomic analysis of senescence-associated genes. New Phytol..

[14] Jukanti A.K., Heidlebaugh N.M., Parrott D.L., Fischer I.A., McInnerney K., Fischer A.M. (2008). Comparative transcriptome profiling of near-isogenic barley (*Hordeum vulgare*) lines differing in the allelic state of a major grain protein content locus identifies genes with possible roles in leaf senescence and nitrogen reallocation. New Phytol..

[15] Cohen M., Hertweck K., Itkin M., Malitsky S., Dassa B., Fischer A.M., Fluhr R. (2022). Enhanced proteostasis, lipid remodeling, and nitrogen remobilization define barley flag leaf senescence. J. Exp. Bot..

[16] Díaz-Mendoza M., Velasco-Arroyo B., González-Melendi P., Martínez M., Díaz I. (2014). C1A cysteine protease-cystatin interactions in leaf senescence. J. Exp. Bot..

[17] Hollmann J., Gregersen P.L., Krupinska K. (2014). Identification of predominant genes involved in regulation and execution of senescence-associated nitrogen remobilization in flag leaves of field grown barley. J. Exp. Bot..

[18] Velasco-Arroyo B., Diaz-Mendoza M., Gandullo J., Gonzalez-Melendi P., Santamaria M.E., Dominguez-Figueroa J.D., Hensel G., Martinez M., Kumlehn J., Diaz I. (2016). HvPap-1 C1A protease actively participates in barley proteolysis mediated by abiotic stresses. J. Exp. Bot..

[19] Cambra I., Martinez M., Dáder B., González-Melendi P., Gandullo J., Santamaría M.E., Diaz I. (2012). A cathepsin F-like peptidase involved in barley grain protein mobilization, HvPap-1, is modulated by its own propeptide and by cystatins. J. Exp. Bot..

[20] Gomez-Sanchez A., Santamaria M.E., Gonzalez-Melendi P., Muszynska A., Matthess C., Martinez M., Diaz I. (2021). Repression of barley cathepsins, HvPap-19 and HvPap-1, differentially alters grain composition and delays germination. J. Exp. Bot..

[21] Frank S., Hollmann J., Mulisch M., Matros A., Carrión C.C., Mock H.P., Hensel G., Krupinska K. (2019). Barley cysteine protease PAP14 plays a role in degradation of chloroplast proteins. J. Exp. Bot..

[22] Hu G., Evans C.P., Satterfield K., Ellberg S., Marshall J.M., Schroeder K.L., Obert D.E. (2024). Registration of ‘GemCraft’ spring malting barley cultivar. J. Plant Regist..

[23] Metodiev M., Demirevskakepova K. (1992). Rubisco quantitation in leaves of different barley varieties by enzyme-linked immunosorbent assay. J. Exp. Bot..

[24] Feller U., Anders I., Mae T. (2008). Rubiscolytics: Fate of Rubisco after its enzymatic function in a cell is terminated. J. Exp. Bot..

[25] Heidlebaugh N.M., Trethewey B.R., Jukanti A.K., Parrott D.L., Martin J.M., Fischer A.M. (2008). Effects of a barley (*Hordeum vulgare*) chromosome 6 grain protein content locus on whole-plant nitrogen reallocation under two different fertilisation regimes. Funct. Plant Biol..

[26] Dann M.S., Pell E.J. (1989). Decline of activity and quantity of ribulose bisphosphate carboxylase/oxygenase and net photosynthesis in ozone-treated potato foliage. Plant Physiol..

[27] Lehnherr B., Grandjean A., Machler F., Fuhrer J. (1987). The effect of ozone in ambient air on ribulosebisphosphate carboxylase/oxygenase activity decreases photosynthesis and grain yield in wheat. J. Plant Physiol..

[28] Landry L.G., Pell E.J. (1993). Modification of Rubisco and altered proteolytic activity in O_3_-stressed hybrid poplar (*Populus maximowizii* x *trichocarpa*). Plant Physiol..

[29] Pell E.J., Eckardt N., Enyedi A.J. (1992). Timing of ozone stress and resulting status of ribulose bisphosphate carboxylase/oxygenase and associated net photosynthesis. New Phytol..

[30] Jukanti A.K., Fischer A.M. (2008). A high-grain protein content locus on barley Hordeum vulgare chromosome 6 is associated with increased flag leaf proteolysis and nitrogen remobilization. Physiol. Plant..

[31] Twining S.S. (1984). Fluorescein isothiocyanate-labeled casein assay for proteolytic enzymes. Anal. Biochem..

[32] Matsumoto K., Mizoue K., Kitamura K., Tse W.C., Huber C.P., Ishida T. (1999). Structural basis of inhibition of cysteine proteases by E-64 and its derivatives. Biopolymers.

[33] Yoon M.C., Phan V., Podvin S., Mosier C., O’Donoghue A.J., Hook V. (2023). Distinct cleavage properties of cathepsin B compared to cysteine cathepsins enable the design and validation of a specific substrate for cathepsin B over a broad pH range. Biochemistry.

[34] Hulkower K.I., Butler C.C., Linebaugh B.E., Klaus J.L., Keppler D., Giranda V.L., Sloane B.F. (2000). Fluorescent microplate assay for cancer cell-associated cathepsin B. Eur. J. Biochem..

[35] Zhou W.W., You B.Q., Zheng Y.F., Si S.Y., Li Y., Zhang J. (2024). Expression, purification, and biological activity evaluation of cathepsin L in mammalian cells. Biosci. Biotechnol. Biochem..

[36] Barrett A.J., Kirschke H. (1981). Cathepsin-B, cathepsin-H, and cathepsin-L. Methods Enzymol..

[37] Schulte J., Stöckermann M., Gebhardt R. (2020). Influence of pH on the stability and structure of single casein microparticles. Food Hydrocoll..

[38] Liu Y., Guo R. (2008). pH-dependent structures and properties of casein micelles. Biophys. Chem..

[39] Holwerda B.C., Rogers J.C. (1992). Purification and characterization of aleurain: A plant thiol protease functionally homologous to mammalian cathepsin H. Plant Physiol..

[40] Takahashi K., Ueno T., Tanida I., Minematsu-Ikeguchi N., Murata M., Kominami E. (2009). Characterization of CAA0225, a novel inhibitor specific for cathepsin L, as a probe for autophagic proteolysis. Biol. Pharm. Bull..

[41] Tsuji A., Kikuchi Y., Ogawa K., Saika H., Yuasa K., Nagahama M. (2008). Purification and characterization of cathepsin B-like cysteine protease from cotyledons of daikon radish, *Raphanus sativus*. FEBS J..

[42] Yoon M.C., Christy M.P., Phan V.V., Gerwick W.H., Hook G., O’Donoghue A.J., Hook V. (2022). Molecular features of CA-074 pH-dependent inhibition of cathepsin B. Biochemistry.

[43] Kurinov I.V., Harrison R.W. (1996). Two crystal structures of the leupeptin-trypsin complex. Prot. Sci..

[44] Aoyagi T., Umezawa H. (1981). The relationships between enzyme inhibitors and function of mammalian cells. Acta Biol. Med. Ger..

[45] Desimone M., Krüger M., Wessel T., Wehofsky M., Hoffmann R., Wagner E. (2000). Purification and characterization of an aminopeptidase from the chloroplast stroma of barley leaves by chromatographic and electrophoretic methods. J. Chromatogr. B Anal. Technol..

[46] Tao L., Zhou H., Guo X.S., Long R.J., Zhu Y., Cheng W. (2011). Contribution of exopeptidases to formation of nonprotein nitrogen during ensiling of alfalfa. J. Dairy Sci..

[47] Martínez D.E., Bartoli C.G., Grbic V., Guiamet J.J. (2007). Vacuolar cysteine proteases of wheat (*Triticum aestivum* L.) are common to leaf senescence induced by different factors. J. Exp. Bot..

[48] van der Hoorn R.A.L., Leeuwenburgh M.A., Bogyo M., Joosten M.H.A.J., Peck S.C. (2004). Activity profiling of papain-like cysteine proteases in plants. Plant Physiol..

[49] Daina A., Michielin O., Zoete V. (2014). iLOGP: A simple, robust, and efficient description of n-octanol/water partition coefficient for drug design using the GB/SA approach. J. Chem. Inf. Model..

[50] Pungercar J.R., Caglic D., Sajid M., Dolinar M., Vasiljeva O., Pozgan U., Turk D., Bogyo M., Turk V., Turk B. (2009). Autocatalytic processing of procathepsin B is triggered by proenzyme activity. FEBS J..

[51] Gombert J., Etienne P., Ourry A., Le Dily F. (2006). The expression patterns of *SAG12/Cab* genes reveal the spatial and temporal progression of leaf senescence in *Brassica napus* L. with sensitivity to the environment. J. Exp. Bot..

[52] Zmienko A., Samelak-Czajka A., Goralski M., Sobieszczuk-Nowicka E., Kozlowski P., Figlerowicz M. (2015). Selection of reference genes for qPCR- and ddPCR-based analyses of gene expression in senescing barley leaves. PLoS ONE.

[53] James M., Poret M., Masclaux-Daubresse C., Marmagne A., Coquet L., Jouenne T., Chan P., Trouverie J., Etienne P. (2018). SAG12, a major cysteine protease involved in nitrogen allocation during senescence for seed production in *Arabidopsis thaliana*. Plant Cell Physiol..

[54] Grbic V. (2003). SAG2 and SAG12 protein expression in senescing Arabidopsis plants. Physiol. Plant..

[55] Rogers J.C., Dean D., Heck G.R. (1985). Aleurain: A barley thiol protease closely related to mammalian cathepsin H. Proc. Natl. Acad. Sci. USA.

[56] Poret M., Chandrasekar B., van der Hoorn R.A.L., Avice J.-C. (2016). Characterization of senescence-associated protease activities involved in the efficient protein remobilization during leaf senescence of winter oilseed rape. Plant Sci..

[57] Eason J.R., Ryan D.J., Watson L.M., Hedderley D., Christey M.C., Braun R.H., Coupe S.A. (2005). Suppression of the cysteine protease, aleurain, delays floret senescence in *Brassica oleracea*. Plant Mol. Biol..

[58] Griffiths C.M., Hosken S.E., Oliver D., Chojecki J., Thomas H. (1997). Sequencing, expression pattern and RFLP mapping of a senescence-enhanced cDNA from Zea mays with high homology to oryzain gamma and aleurain. Plant Mol. Biol..

[59] Li Q., Bettany A.J.E., Donnison I., Griffiths C.M., Thomas H., Scott I.M. (2000). Characterisation of a cysteine protease cDNA from *Lolium multiflorum* leaves and its expression during senescence and cytokinin treatment. Biochim. Biophys. Acta.

[60] Richau K.H., Kaschani F., Verdoes M., Pansuriya T.C., Niessen S., Stüber K., Colby T., Overkleeft H.S., Bogyo M., Van der Hoorn R.A.L. (2012). Subclassification and biochemical analysis of plant papain-like cysteine proteases displays subfamily-specific characteristics. Plant Physiol..

[61] Greenbaum D., Medzihradszky K.F., Burlingame A., Bogyo M. (2000). Epoxide electrophiles as activity-dependent cysteine protease profiling and discovery tools. Chem. Biol..

[62] Nickerson J.L., Doucette A.A. (2020). Rapid and quantitative protein precipitation for proteome analysis by mass spectrometry. J. Proteome Res..

[63] Laemmli U.K. (1970). Cleavage of structural proteins during the assembly of the head of bacteriophage T4. Nature.

[64] Daina A., Michielin O., Zoete V. (2017). SwissADME: A free web tool to evaluate pharmacokinetics, drug-likeness and medicinal chemistry friendliness of small molecules. Sci. Rep..

