# Supplementary material for: Cathepsin B- and L-like Protease Activities Are Induced During Developmental Barley Leaf Senescence
Source: Plants (Basel). 2024 Oct 28;13(21):3009. doi: 10.3390/plants13213009 (PMC11548477; doi:10.3390/plants13213009)
Supplement: Supplementary file 1 [file plants-13-03009-s001.zip › plants-3269640-supplementary.pdf]

Supplementary Materials

Cathepsin B- and L-like Protease Activities Are Induced During Developmental Barley Leaf Senescence

Igor A. Schepetkin * and Andreas M. Fischer *

Department of Plant Sciences and Plant Pathology, Montana State University, Bozeman, MT 59717, USA

* Correspondence: igor@montana.edu (I.A.S.); fischer@montana.edu (A.M.F.); Tel.: +1-406-994-5908 (A.M.F.)

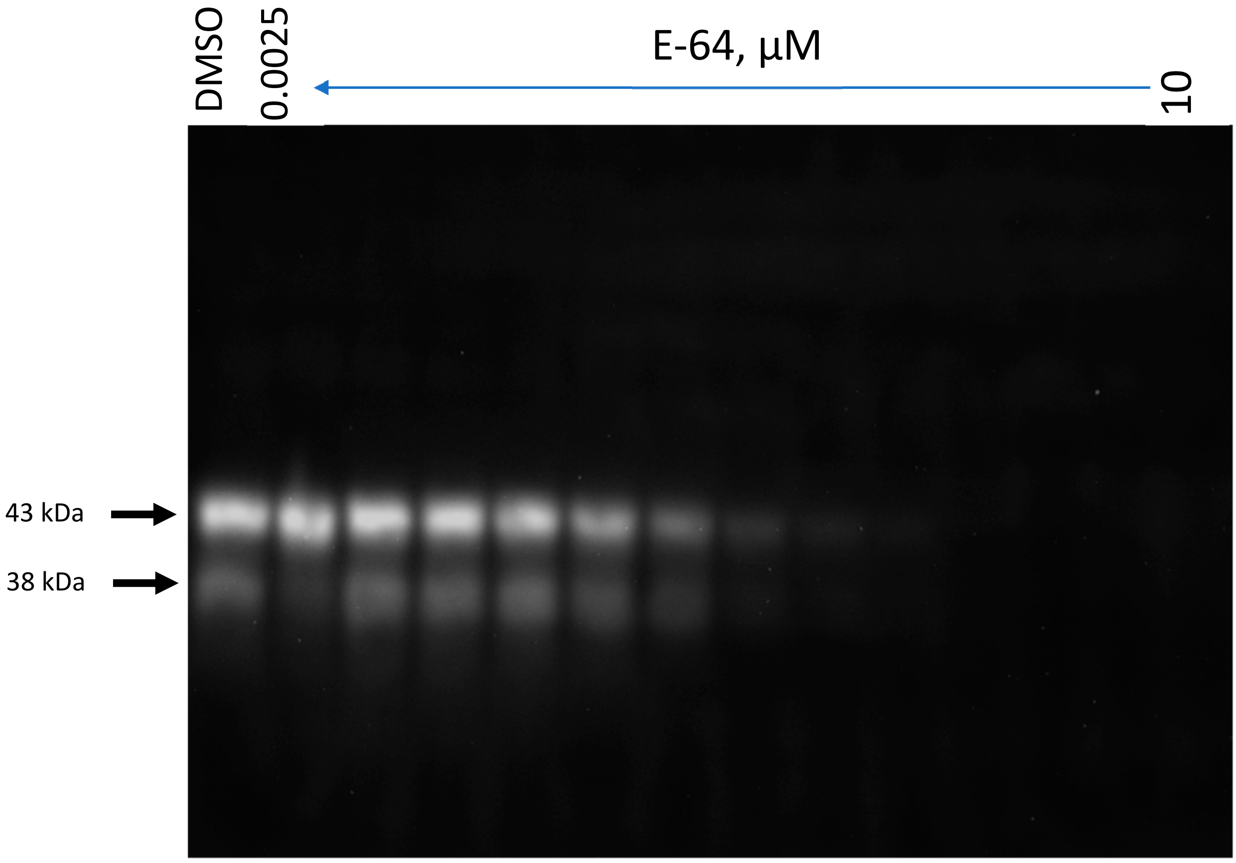

Supplementary Figure S1. Concentration-dependent effect of the protease inhibitor E-64 on DCG-04 binding to the 38- and 43-kDa bands. The protein extracts of barley leaves obtained 4 weeks after flowering were preincubated with different concentrations of the inhibitor and proteins were labeled with 2.5 µM DCG-04 at pH 5.5 for 3 h. DMSO (solvent for inhibitors) was used as a control. For the treatment, 2-fold dilutions of E-64 were used (10, 5, 2.5, 1.25, 0.64, 0.32, 0.16, 0.08, 0.04, 0.02, 0.01, 0.005, and 0.0025 µM). Representative blot from three independent experiments.

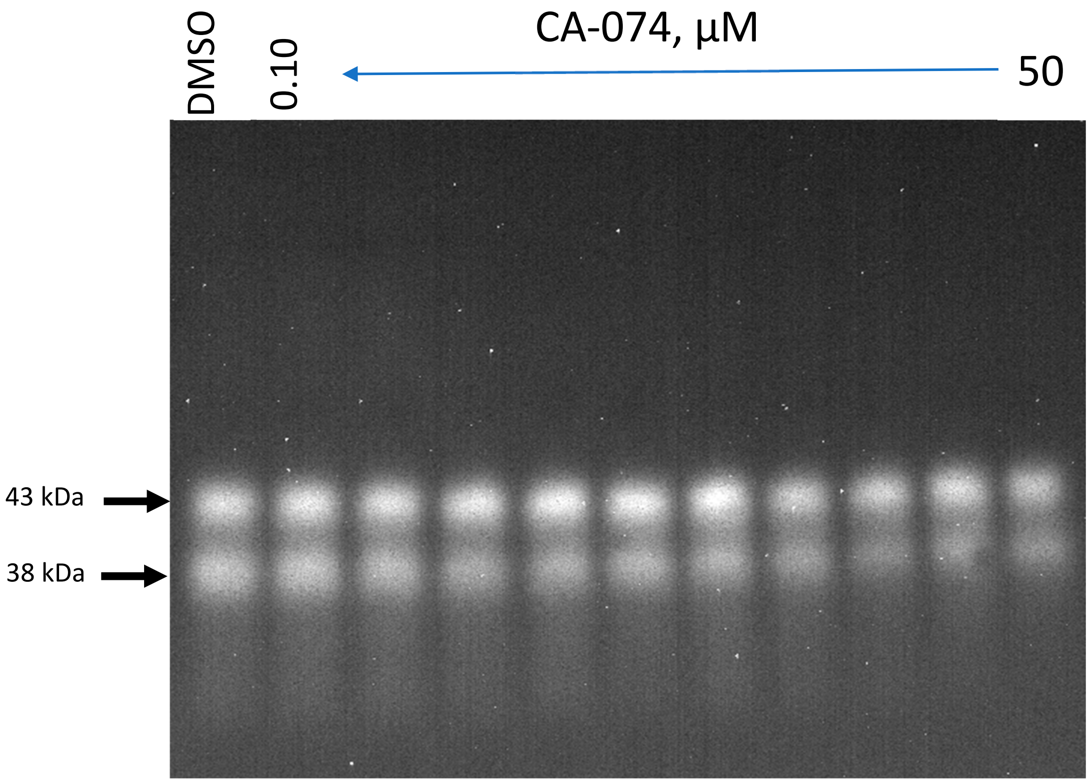

Supplementary Figure S2. Concentration-dependent effect of the protease inhibitor CA-074 on DCG-04 binding to the 38- and 43-kDa bands. The protein extracts of barley leaves obtained 4 weeks after flowering were preincubated with different concentrations of the inhibitor and proteins were labeled with 2.5 µM DCG-04 at pH 5.5 for 3 h. DMSO (solvent for inhibitors) was used as a control. For the treatment, 2-fold dilutions of CA-074 were used (50, 25, 12.5, 6.4, 3.2, 1.6, 0.8, 0.4, 0.2, and 0.1 µM). Representative blot from three independent experiments.

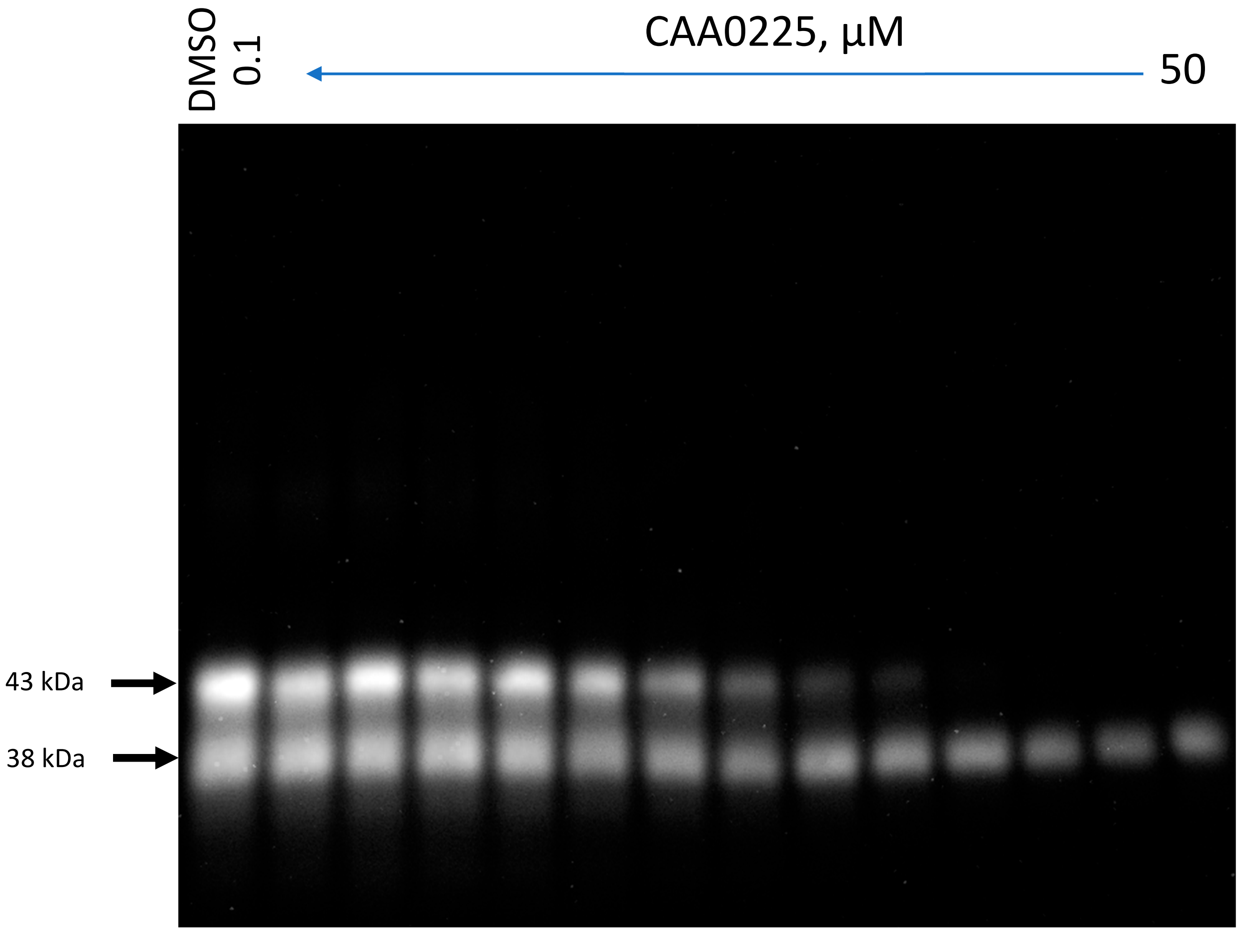

Supplementary Figure S3. Concentration-dependent effect of the protease inhibitor CA0225 on DCG-04 binding to the 38- and 43-kDa bands. The protein extracts of barley leaves obtained 4 weeks after flowering were preincubated with different concentrations of the inhibitor and proteins were labeled with 2.5 µM DCG-04 at pH 5.5 for 3 h. DMSO (solvent for inhibitors) was used as a control. For the treatment, 2-fold dilutions of CA0225 were used (50, 25, 12.5, 6.4, 3.2, 1.6, 0.8, 0.4, 0.2, and 0.1 µM). Representative blot from two independent experiments.
